# Trends in ECT (Electroconvulsive Therapy) Utilization During Pregnancy and Post-Partum Period: National Inpatient Sample 2002-2015

**DOI:** 10.1192/j.eurpsy.2022.1904

**Published:** 2022-09-01

**Authors:** S. Jaiswal, C. Trivedi, K. Shah, A. Bishay Elshokiry, M. Adnan, F. Tazin, Z. Mansuri

**Affiliations:** 1 University of Texas Southwestern Medical Center Dallas, Psychiatry, Dallas, United States of America; 2 Texas Tech University Health Science Center, Psychiatry, Midland, United States of America; 3 Griffin Memorial Hospital, Psychiatry, Norman, United States of America; 4 North Park University, Research, Chicago, United States of America; 5 University of Toranto, Psychiatry, Toranto, Canada; 6 East Liverpool Hospital, Psychiatry, East Liverpool, United States of America; 7 Boston Childrens Hospital/Harvard Medical School, Child And Adolescent Psychiatry, Brighton, United States of America

**Keywords:** ECT, Psychiatric comorbidities, pregnant, Post-Partum Period

## Abstract

**Introduction:**

The use of Electroconvulsive therapy (ECT) during pregnancy and in the post-partum period is a critical decision for both providers and their patients. ECT utilization during this critical period needs to be better understood to assess the need and allocate resources for this valuable treatment option.

**Objectives:**

1) To evaluates baseline characteristics and analyze ECT utilization trends for pregnant and post-partum patients hospitalized in the US. 2) To provide insight into ECT use among inpatient pregnant women with different age groups with various comorbid psychiatric disorders.

**Methods:**

The study used the 2002-2015 National (Nationwide) Inpatient Sample (NIS) data. Descriptive statistical and trend analyses were conducted to evaluate data.

**Results:**

A study found that a total of 924 pregnancy-related hospitalizations required ECT treatment; 92.2% of these ECTs were conducted in urban hospitals. The mean age of women was 30.3 years, and the majority (71%) were of the White race. Mood disorders (major depressive disorder- 51.9% and bipolar disorder- 37.9%) accounted for the most common comorbid psychiatric illnesses. The payer source (Medicare/Medicaid vs. Private Insurance) was almost equal (47.9 vs. 46.8). Though not statistically significant, the trend analysis showed that the proportion of ECTs during pregnancy out of the total ECT performed for the year almost doubled (0.24% to 0.47%) from 2008 to 2015.

**Conclusions:**

Though not statistically significant, the use of ECT in pregnant women has increased in 2015 compared to 2002. Results will help clinicians, policymakers, and various stakeholders to optimize ECT utilization, reimbursement and ultimately improve clinical outcomes.

**Disclosure:**

No significant relationships.

